# Comparing audiological evaluation and screening: a study on presbycusis

**DOI:** 10.1590/S1808-86942011000100012

**Published:** 2015-10-19

**Authors:** Alessandra Giannella Samelli, Camila Aparecida Negretti, Kerli Saori Ueda, Renata Rodrigues Moreira, Eliane Schochat

**Affiliations:** 1 PhD in Sciences - Medical School of the University of São Paulo -FMUSP, Professor - Department of Physical Therapy, Speech and Hearing Therapy and Occupational Therapy - FMUSP; 2 Speech and Hearing Therapist graduated from FMUSP; 3 Speech and Hearing Therapist graduated from FMUSP; 4 PhD in Sciences - FMUSP, Speech and Hearing Therapist - FMUSP; 5 Associate Professor - FMUSP, Associate Professor - Department of Physical Therapy, Speech and Hearing Therapy and Occupational Therapy - FMUSP

**Keywords:** audiometry, hearing, aged, hearing loss, presbycusis

## Abstract

Given the high prevalence of presbycusis and the damage it brings about, a screening test can be useful in the identification of hearing loss in primary care.

**Aim:** To estimate the prevalence of hearing loss in a representative sample of elderly people living at Butantan using an audiological screening method (questionnaire) and a basic audiological evaluation; to compare the results of the two kinds of evaluations, checking the validity of this tool for hearing loss screening.

**Design:** Cross sectional descriptive study.

**Materials and Methods:** 200 individuals (above 60 years old, both genders) were randomly selected to undergo audiological screening (questionnaire). Another randomly selected group encompassed 100 individuals who were submitted to a set of audiological tests. Then, we compared the results from the two methods.

**Results:** There were no statistically significant associations between the questionnaire and the degree of hearing loss of the patients.

**Conclusion:** The prevalence of hearing loss in our sample was of 56% in the screening and of 95% when checked by the audiological evaluation. Therefore, screening was not proven valid to assess hearing when compared to audiological evaluation.

## INTRODUCTION

Today, Brazil is going through a period of growth of its elderly population. According IBGE projections for 2028, we are expected to have 38 million people with more than 60 years of age^1^, in other words, there will be more than a double-fold increase in the current elderly population.

This populational growth will bring about an even greater demand for public health policies, especially in terms of diagnosis and treatment^2^.

One of the implications of this boom in this population will be a likely increase in presbycusis, which is an aging-related type hearing loss. Its etiology may have consequences which stem from a number of insults inflicted throughout one's life, including aging-related degeneration, noise exposure, ototoxic agents and otological agents, in general. Presbycusis can be considered whenever, in the absence of other etiologies, there is bilateral, symmetrical, insidious hearing loss, especially in the high frequencies, however it can affect all hearing frequencies, in a population older than 40 years. It is highly influenced by genetics, diet and systemic diseases, and it may affect 30 to 60% of the geriatric population, and prevalence will be greater or lower in relation to socioeconomic variables^3^, ^4^, ^5^, ^6^, ^7^, ^8^.

Hearing loss acquired during adulthood can impair quality of life and the person's integration to society. Because of the hearing loss, many individuals change their life structures, bringing about professional and affection losses, and it may also negatively impact relationships with friends and families^3,^^9,^^10^. These psychological and social issues stemming from hearing loss are called handicap or disadvantage by the World Health Organization^11,^^12^.

The psychological impact stemming from the hearing loss can be assessed by means of self-assessment questionnaires, which are useful tools used to quantify the emotional and social consequences perceived in function of the hearing loss. These instruments may be used in different clinical routines, such as screening and/or initial interviews, assessment of the sound amplification device and assessment of the auditory rehabilitation programs, providing the audiologist the means to understand the impact of this handicap in the elderly and the needs of this population^12^, ^13^, ^14^.

Because of the high prevalence of presbycusis and the handicap it causes, a screening may be useful to identify the hearing loss in primary care. Although its importance is clear, especially in developing countries, the screening methods vary considerably in terms of strategies, techniques, application and effectiveness. Audiometry is the gold standard test to assess hearing loss; nonetheless, it can be difficult in some places because of problems associated with access, reference and reimbursement. For these reasons, many clinicians rely on self-deployed questionnaires as means to screen for hearing loss, since they are fast and inexpensive instruments^15,^^16^.

Thus, the goals of the present study were:

1) To estimate the hearing loss prevalence in the elderly who live in the Health District of Butantã using the audiological screening and basic audiologic evaluation;
2) To compare the audiological screening results and that of basic audiological evaluation, checking the validity of such instrument to be used as hearing screening tool.

## MATERIALS AND METHODS

The study was carried out within the standards required by the Declaration of Helsinki and it was approved by the Ethics in Research Committee of our Institution (#1024). This institution is a reference hospital for those individuals who live in the geographic area covered by the Health District of Butantã, with an estimated population of about 400,000 people.

We carried out a cross-sectional cohort study, using the sample defined for the study: “A epidemiologia da demência e doença de Alzheimer em populações miscigenadas no Brasil e em Cuba”, also carried out by this institution.

For sample selection purposes, we selected the areas covered by the hospital - those belonging to the administrative districts of Butantã, Rio Pequeno and Raposo Tavares, broken down into two groups:

- Censused sectors which have slums, such as São Remo, São Domingos, Sapé, Vila Dalva and others. The Jardim São Remo slum started in an area which belonged to the University of São Paulo about 30 years ago. It has a relatively stable population, with well-defined streets and houses with numbers, it has been counted in recent censuses and they have electrical power and running water. The remaining is more recent and some are in risky areas. Besides socio-economic deprivations, the residents of these slums have high likelihood of having little or no formal education at all. They are mostly domestic migrants, who lived the beginning of their lives in poor areas of the country;
- Censured sectors of residential areas from the districts of Butantã, Rio Pequeno and Raposo Tavares, defined at random according to socio-economic metrics. The collection areas were numbered by household in order to register the residents with 65 years of age or more. All the co-residents of elderly patients were also identified. All the residents with more than 60 years of age from the selected censused sectors were recruited. The total number of participants was 2,000. Those individuals aged 60 years or more were considered elderly, based on the criterion established by the World Health Organization, which establishes this age range as the beginning of the elderly age in developed countries.

We randomly selected 200 individuals from both genders, at the age range starting from 60 years, among all the participants in the study: “A epidemiologia da demência e doença de Alzheimer em populações miscigenadas no Brasil e em Cuba”.

Of the 200 initially selected individuals, fifteen could not take part in the study. The causes for not participating were: cognitive deficit which prevented them from understanding the questions (7 cases); refusal to answer the questionnaire (4 cases); impossibility of applying the entire questionnaire because of lack of contact with family member and/or caretakers, which should answer the last question (4 cases). Thus, the series was formed by 185 individuals who underwent the screening.

For the second stage of the present study, we selected again 100 from the 200 previously selected individuals, half from each gender. Of these 100, 10 previously selected individuals could not undergo complete audiological evaluation. The causes were: unanswered questionnaire (2 cases); the individual did not feel well during the test (3 cases) and did not understand the orders given during the assessment (4 cases). Thus, in this stage, 91 individuals participated.

Only those individuals who agreed with participated in the study, and they signed the free and informed consent form (either the individual or a guardian signed it).

We then applied a questionnaire (audiological *screening*) with 15 questions, concerning the different day-today communication situations and hearing difficulties, as well as social isolation, which aimed at assessing the impact caused by the hearing loss to the elderly's life. This questionnaire is used in the hospital's audiology department, and it was created based on the *Nursing Home Hearing Handicap -* NHHI^17^, *Hearing Handicap Inventory for the Elderly*^18^, and *Hearing Handicap Inventory for Adults -* HHIA^19^ questionnaires. Based on the questionnaire, the answers were scored as follows: 0 - Never; 1 - Occasionally; 2 - In half of the time; 3 - Always.

The questions were answered by the elderly himself and only one question was asked to family members (the family member's impression on the elderly's hearing difficulties). The answers from each questionnaire were added up and we then we obtained the final score for each individual. Based on this result, the individuals were broken down into three groups. For each group we associated a handicap perception based on the performance analysis criterion of the individuals assessed by the screening process according to:

- Group 1: 0 to 10 points (no handicap perception);
- Group 2: 11 to 20 points (mild/moderate handicap perception);
- Group 3: 21 to 45 points (severe/significant handicap perception).

During audiological evaluation, we initially studied the hearing thresholds obtained for frequencies 250 to 8,000 Hz through air conduction and, when these thresholds went beyond 20 dB HL, they were also determined by bone conduction in the frequencies of 500 to 4,000 Hz. The hearing thresholds were classified in degrees, according with the Lloyd and Kaplan^20^criterion, which considers thresholds up to 25 dBHL, hearing loss from 26 and 40 dBHL, moderate between 41 and 55 dBHL, moderately severe between 56 and 70 dBHL, severe between 71 and 90 dBHL and profound those thresholds higher than 90 dBHL.

Following that, we studied the speech detection threshold and we obtained the speech intelligibility curve. For these procedures we used the Madsen audiometer - model Midimate 622.

We also did tympanometry and ipsi and contralateral acoustic reflex studies, using the GSI middle ear analyzer - Tympstar model.

We used the chi-square tests for Independence and Anova, as well as the confidence interval approach. The significance level assigned was of 0.05 (5%).

## RESULTS

For the 185 individuals who answered the questionnaire (auditory screening), the groups were broken down according to the score obtained:

- Group 1 - 107 individuals (57.8%);
- Group 2 - 43 individuals (23.2%);
- Group 3 - 35 individuals (19%).

For the 91 individuals who also underwent audiologic assessment, the groups were broken down according to the score obtained in the questionnaire, and they were distributed as follows:

- Group 1 - 40 individuals (43.95%);
- Group 2 - 29 individuals (31.85%);
- Group 3 - 22 individuals (24%).

Table 1 shows a better characterization of the score obtained for the 3 groups, in the case of the 91 individuals.

**Table 1:** Confidence Interval for the score obtained in the questionnaire, considering the 91 individuals, broken down by group.

Questionnaire	Group 1	Group 2	Group 3
Mean	4,38	15,07	25,64
Median	4	15	27
Standard Deviation	2,84	2,79	7,15
Minimum	0	11	21
Maximum	10	20	35
Size	40	29	22
Lower limit	3,49	14,05	22,65
Upper limit	5,28	16,08	28,62

Legend: Statistical confidence interval of 95%.

For the audiological evaluation analysis, we considered the degree of hearing loss according to the worst threshold presented and the results were presented and broken down by groups, according to the result from the questionnaire, for the 91 individuals who participated in this stage of the evaluation. The results are described on Table 2.

**Table 2:** Percentage of individuals per degree of hearing loss in the different groups.

	Group 1 (n = 40)	
Degree of hearing loss	RE involvement (%)	LE Involvement (%)
No loss	0	15
Mild	22,5	25
Moderate	52,5	47,5
Mod. Severe	2,5	2,5
Severe	10	7,5
Profound	12,5	2,5
Total	100	100
	Group 2 (n = 29)	
Degree of loss	RE involvement (%)	LE Involvement (%)
No loss	3,5	7
Mild	20,6	13,7
Moderate	37,9	37,9
Mod. Severe	7	10,3
Severe	31	27,6
Profound	0	3,5
Total	100	100
	Group 3 (n = 22)	
Degree of loss	RE involvement (%)	LE Involvement (%)
No loss	0	4,5
Mild	9,1	18,2
Moderate	50	36,4
Mod. severe	9,1	9,1
Severe	22,7	22,7
Profound	9,1	9,1
Total	100	100

Through the results obtained from the audiological evaluation we can notice that in the three groups, most of the individuals had moderate hearing loss. The second largest occurrence in group 1 was the mild hearing loss and in groups 2 and 3 it was the severe hearing loss.

We can also notice that only 10 of the 182 ears did not have any degree of hearing loss, in other words, 5.5% of the ears.

As to the type of hearing loss, there were no cases of conductive hearing loss and only 4 cases of mixed hearing loss. The other hearing loss cases were of the sensorineural type (93.4%).

Based on the results presented on Table 3, we noticed that there were no statistically significant association between the complaint - collected from the questionnaire, and the hearing loss degree in the right and/or left ears. Nonetheless, we can say that there was a tendency towards variable association for the right ear (*p* = 0.085).

**Table 3:** Percentage of individuals distributed according to the groups and auditory thresholds for the right ear (RE) and left ear (LE).

RE hearing thresholds	Complaint reported in the questionnaire					
		Group 1 (%)	Group 2 (%)	Group 3 (%)	Total (n)	Total (%)
No loss		0%	3,5%	0%	1	1,09%
Mild		22,5%	20,6%	9,1%	17	18,6%
Moderate		52,5%	37,9%	50%	43	47,3%
Mod/Severe		2,5%	7%	9,1%	5	5,5%
Severe		10%	31%	22,7%	18	19,8%
Profound		12,5%	0%	9,1%	7	7,7%
Total		43,95%	31,85%	24 %	91	100%
LE hearing thresholds	Complaint reported in the questionnaire					
		Group 1 (%)	Group 2 (%)	Group 3 (%)	Total (n)	Total (%)
No loss	6	15%	7%	4 %	9	10 %
Mild	10	25%	13,8%	18 %	18	19,8 %
Moderate	19	47,5%	37,9%	36,4%	38	41,8 %
Mod/Severe	1	2,5%	10,3%	10 %	6	6,6 %
Severe	3	7,5%	27,6%	22,7%	16	17,5 %
Profound	1	2,5%	3,5%	9 %	4	4,3 %
Total	40	43,95%	31,85%	24 %	91	100%

Legend: Chi-square test - p-value = 0.085# (since it is near the acceptance level, it is considered as tending towards significance) for the right ear and p-value = 0.355 for the left ear.

Based on Table 4, we can say that for those individuals who did not complain of the handicap (degree 1) in the questionnaire, the likelihood of that person having hearing loss was of 92.5%. Now, for individuals with significant perception of the *handicap* (degree 3), the likelihood of the person having hearing loss was of 100%.

**Table 4:** Likelihood of hearing loss associated with the score obtained from the questionnaire.

	Normal hearing	Hearing loss	Hearing loss likelihood
Degree 1	3	37	92,50%
Degree 2	1	29	96,66%
Degree 3	0	21	100%
Total	4	87	--

We carried out a new comparison, associating the mean value of the score obtained from the questionnaires and the hearing thresholds. To do that, the individuals were grouped according to hearing thresholds in the worst ear, in other words, Group A (normal hearing thresholds), Group B (mild hearing loss), Group C (moderate hearing loss) and Group D (moderately severe to profound hearing loss). The results are depicted on Table 5 and they indicate statistical significance in regards of the mean value obtained from the questionnaire, since the auditory thresholds are worse.

**Table 5:** Mean and standard deviation of the score obtained from the questionnaire per group, according to auditory thresholds.

Group	Mean	Standard deviation	n
A	4,8	6,3	4
B	7,1	9,9	11
C	12,2	9,3	45
D	17,4	8,2	31
p = 0,002*			

Legend: Anova

## DISCUSSION

In regards of the perception of the handicap, checked in the questionnaire, we noticed that the majority of individuals studied (approximately 56%) reported they perceived it, which is in agreement with those findings from Pinzan-Faria and Iório^12^, who used the HHIE, and found more than 75% of the series perceiving a mild to severe handicap. Melo et al.^10^ and Nóbrega et al.^21^ used the HHIE abridged version, and reported that only 21.27% and 23% of the elderly assessed felt social and/or emotional disadvantages arising from their hearing loss, respectively. One of the justifications for this difference between the results of this study is associated with the variability in perception of the hearing loss consequences and the life style the elderly has in his/her community, which directly reflects the diversity found in the scores from the questionnaires^10,^^12^, and they may also be influenced by the different instruments used.

The prevalence of hearing loss, studied by the basic audiological evaluation in this study was very high, in other words, around 95%, which is different from similar studies, in which the prevalence was around 60 to 70%^3,^^8,^^10^. Valete-Rosalino and Rozenfeld^16^ ran a literature review about presbycusis, and found values varying between 2 and 83% in relation to the prevalence of hearing loss in numerous studies, which used different classification criteria. Thus, the use of lower intensities as a cutting point, including higher frequencies as well as the use of the worst ear to define hearing loss result in an increase in hearing loss prevalence, which was the study at hand, because of these more encompassing criteria (lower intensity, high frequencies and classification by the worst ear).

In regards of the audiological profile of the population studied, we noticed the prevalence of bilateral sensorineural hearing loss, of descending shape. Such findings have also been reported by Melo et al.^10^; Mattos and Veras^8^; Baraldi et al.^3^.

As to the degree of hearing loss, we noticed a predominance of moderate hearing loss, and the mild hearing loss type was the second largest occurrence in Group 1; severe hearing loss was the second highest occurrence in Groups 2 and 3. These results differ from those of other similar studies, since most report a higher prevalence of mild hearing loss^3,^^10,^^12^. This fact has also been associated with the use of more encompassing hearing loss classification critiera^16^, as previously discussed.

In the analysis which compared the complaint stated in the questionnaire and the degree of hearing loss, there was no statistically significant association for the right and left ears. These data indicate that the individual with a hearing handicap may not show hearing loss necessarily, or not have such a severe hearing loss as stated in the questionnaire. The opposite is also possible, in other words, individuals who do not complain of hearing loss in the questionnaire, have hearing loss of different degrees. These observations were also made by other authors^12,^^14,^^21^, indicating that there is no direct relationship between the degree of hearing loss and the handicap, since elderly patients with different degrees of hearing loss may have the same handicap.

In order to check whether the questionnaire used was sensitive as an only method for auditory screening, we compared the likelihood of hearing loss with the degrees obtained through the handicap quantification done by the questionnaire. This analysis revealed a very high number of false-negative results, considering those patients who did not perceive a handicap (degree 1). If we consider only the elderly who had degrees 2 and 3 of hearing loss as per reported in the questionnaire, the instrument's sensitivity proved to be higher than 96%. Nonetheless, in this study, as per previously mentioned, there was a very small number of individuals without hearing loss, and this has impaired more specific conclusions on this aspect. Notwithstanding, this instrument alone was not feasible for hearing screening because of a large number of false-positive, which matches the findings from Pinzan-Faria and Iório^12^.

And finally, the last analysis made tried to study whether a worsening in auditory thresholds had any relation with the mean score obtained in the questionnaire. This relation proved positive, in other words, as the elderly got worse in their auditory sensitiveness, there was an increase in their self-perception of the handicap, proven by a worsening in the questionnaire scores, per group, and this was also reported by Pinzan-Faria and Iório^12^, despite the large individual variability in the questionnaire score, indicating that there is no direct relation between the degree of hearing loss and the handicap, as mentioned above.

The growth in the number of elderly people in the population will increase even further the demand for public health policies, especially in regards of presbycusis. Thus, it is urgent to establish the guidelines to develop diagnostic programs, individual sound amplification devices and a specific program of auditory reeducation for the elderly with hearing loss, so as to prevent them from suffering the psychological and social losses stemming from the hearing impairment^2,^^14^. Studies concerning hearing loss prevalence and screening and/or diagnostic methods, as well as methods to quantify the auditory handicap and to follow up the auditory rehabilitation process are fundamental.

The present study reported that the questionnaire utilized is not a method to be used alone in auditory screening; however, it indicates the degree of hearing loss for the elderly, which is highly variable, regardless of the hearing loss level, since it depends on social and psychological adaptation factors, as well as on factors associated with age and physical health^12^, ^13^, ^14^. Thus, the use of questionnaires is important, since it completes the data brought about by audiological evaluation, providing hints on the elderly functional capacity and the psychosocial impact of the hearing loss for the elderly, which cannot be totally predicted from the audiogram.

## CONCLUSION

1) The hearing loss prevalence found in this population was approximately 56% according to the *screening* and 95% according to basic audiological evaluation.

2) The *screening* (questionnaire) did not prove to be a valid instrument to be used in auditory screening, when compared to basic audiological evaluation.
